# Poor-Law Infirmary—Publications in 1912

**Published:** 1913-04-05

**Authors:** 


### POOR-LAW INFIRMARY—PUBLICATIONS IN 1912.

Not many important publications dealing with matters of interest to those concerned in the administration of Poor-Law infirmaries as such were issued during the year 1912. The chief of them are referred to below:

### The Forty-first Annual Report of the Local Government Board (1911-1912).

The report contains the usual tables showing the number of paupers in receipt of relief on July 1, 1911, and January 1, 1912. The most striking feature brought out by them is the great reduction which has occurred of recent years in the number of paupers over seventy. On January 1, 1910, the number was 195,944; on January 1, 1912, it had sunk to 58,858. As the report points out, this reduction is undoubtedly due to the operation of the Old Age Pensions Act. It is interesting to note that only 11.5 per cent. of all the paupers in receipt of relief on January 1, 1912, were described as able-bodied persons in health. The report contains a short digest of a special return obtained on November 4, 1911, showing the number of inmates in Poor-Law institutions under medical treatment on that day. The number for England and Wales was 97,926, of whom 39,976 were in the sick wards of workhouses and 33,717 in separate institutions for the sick.

The returns obtained from the different Boards of Guardians gave the number of sick classified as acute and chronic medical cases and acute and chronic surgical cases, and it is to be regretted that the numbers so obtained are not included in the report. The table showing the total number of paupers relieved on July 1 and January 1 for each year since 1887 brings out the interesting fact that, while the total number of men in receipt of indoor relief on July 1, 1911, has increased by nearly 100 per cent. when compared with July 1, 1887, the increase in the case of the women is only about 50 per cent. It would seem as though there was some influence—probably the legislation dealing with the compensation of injured workmen—which is tending to render it more and more difficult for all but the most able-bodied men to obtain an independent livelihood. The report shows that the total amount spent in the relief of the poor for the year ended March 1911 was £15,023,130, of which £1,016,492 was spent in "medical relief," apart from the maintenance of the sick. The report states that the Local Government Board issued orders in six more Unions relieving the workhouse master and matron of the duties of visiting the sick wards, and making the superintendent nurse responsible for the cleansing, warming, and ventilation of these wards and for arranging that the inmates are properly cared for and attended. Similar orders have now been issued in a large number of Unions, and this shows how retrograde the draft of the Poor-Law Institutions Order was in its provisions upon this point.

### Report of National Conference on Prevention of Destitution, 1912.*

The report includes papers read in the Public Health Section of the Conference on "The Medical Treatment of Sick Children under School Age," "The Medical Treatment of School Children," and "The Medical Treatment of the Uninsured Sick." One of the most important points brought out in the papers was the immense amount of sickness which occurs amongst children without receiving any medical treatment. Dr. Boobbyer asserted that 80 to 90 per cent. of the children found to be suffering from illness or abnormalities by the school medical officers had never received any medical attention, although the conditions found were such as urgently needed treatment. The papers and the discussion upon them suffered from the fact that the speakers were connected almost entirely with the Public Health Service, and were apparently ignorant of the work Boards of Guardians are already doing in the treatment of children brought to them by their parents as the result of the examination of the school medical officer and of the powers Guardians possess to deal with all cases of illness when the sufferers cannot obtain adequate medical attention, although they may not be destitute in the ordinary sense of the word.

### Poor-Law Reform—A Practical Programme.†

The scheme advocated agrees on broad lines, but with some important modifications, with the proposals of the signatories of the Minority Report of the Royal Commission on the Poor Laws. Boards of Guardians are to be abolished and their duties divided amongst the County Councils, the County Borough Councils, the Urban District Councils, and the Rural District Councils. In the counties the County Councils are to have general supervision of the minor authorities in the area, control and management of all institutions within the county intended for the destitute, such as Poor-Law infirmaries and schools, and some control over finance. The scheme leaves to the Urban and Rural District Councils the administration of all relief in money or kind in the home of the applicant. The mentally defective are to be handed over to an enlarged Lunacy Commission—the County Councils maintaining the institutions—and the institutional relief and treatment of the unemployed is to form part of the duties of the Labour Department of the Board of Trade. In the county boroughs the whole of the Poor-Law work, except as regards the mentally defective and the unemployed, would pass to the Borough Councils. This scheme has much to recommend it. The enlargement of the areas served by the infirmaries and other institutions would permit of a greatly improved classification of the inmates, and would enable specialised treatment to be pro-

* Published by P. S. King and Sons (price 10s. 6d.).
† Published by West Strand Publishing Co. (price 1s.).

vided for the whole of the sick poor in the county area at a reasonable cost. At the same time the relegation of domiciliary relief to the District Councils would ensure that personal and local administration which is so necessary to prevent abuse. There are two points in which the scheme might be improved. The first is that it would be better that the County Councils should take over all the institutions in the county area, including the institutions in county boroughs situated within the county area. In any new system of co-ordination of medical relief, the valuable institutions in the county boroughs should be available for all those within a reasonable radius, whether within or without the boundary of the borough. The more highly specialised institutions must always be provided in the larger towns, and there is no reason why, with improved classification of the inmates and the use of some of the rural institutions as convalescent homes, they should not serve a larger area than they do at present. The second point is this. The scheme suggests that the whole of the institutional Poor-Law medical staff should be placed under the medical officer of health for the county. This is a grave mistake. Medical science has become too complex for one man to be able to deal with both preventive and clinical medicine. The two services should be kept distinct, although there should be full co-operation and interchange of information between them. Both services should be on an equality and responsible through their senior members to the County Council. It is only by keeping the two services thus distinct and equal that it will be possible to maintain a high pitch of efficiency in both.

The Great State—Essays in Construction.‡

This book is an attempt to foreshadow the perfect state where labour is organised to give the maximum effect with the minimum of effort, while the labourer's individuality and independence are increased. Mr. C. J. Bond contributes an interesting chapter on "Health and Healing in the Great State." For the promotion of health in the future he looks to eugenic control of the birth-rate scientifically applied as the result of increasing knowledge and experience, to the increase of leisure permitting of healthy recreation, and to the improvement of housing conditions. He advocates State control for all the voluntary and other agencies dealing with the treatment of the sick and the formation of national medical and nursing services. The whole of the essays are interesting and stimulating and worthy of careful perusal.

‡ Published by Harper and Brothers (price 6s.).

